# Postoperative Necrotizing Enterocolitis Following Inguinal Hernia Repair in an Infant: Case Report and Review of the Literature

**DOI:** 10.7759/cureus.45089

**Published:** 2023-09-12

**Authors:** Rawan Alhalabi, Dalia Belsha, Hala Rabei, Hussein Muad, Heba Farhoud, Ghassan Nakib, Muhammad Eyad Ba'Ath

**Affiliations:** 1 Pediatrics, American Hospital Dubai, Dubai, ARE; 2 Pediatric Gastroenterology, American Hospital Dubai, Dubai, ARE; 3 General Medicine, Damascus University, Damascus, SYR; 4 Pediatric Surgery, Mediclinic Middle East, Dubai, ARE; 5 Pediatric Surgery, University of Sharjah, Sharjah, ARE

**Keywords:** recurrent nec, norovirus, neonate, surgical stress, bowel stricture, inguinal hernia, postoperative necrotizing enterocolitis (nec)

## Abstract

Necrotizing enterocolitis (NEC) following postoperative stress is a rare but life-threatening condition in infants. We report a 3-month-old infant who underwent bilateral inguinal hernia repair and developed NEC. This is the first reported case of an infant developing recurrent NEC with stricture formation after herniotomy. Timely recognition and management are vital due to potentially high mortality rates in severe cases. High index of suspicion is crucial for accurate diagnosis and appropriate management.

## Introduction

Necrotizing enterocolitis (NEC) is the most common life-threatening emergency affecting neonates [1]. It is defined as inflammation of the intestine leading to bacterial invasion and cellular damage, with the risk of stricture formation and bowel perforation [1]. Generally, it presents with poor feeding, vomiting, abdominal distention, and blood in the stool [1-3]. It could be treated conservatively by bowel rest, intravenous broad-spectrum antibiotics, and gastric decompression with a nasogastric tube, and other supportive measures [1,4]. Surgical intervention is indicated if medical management has failed or complications have developed [1,5]. NEC frequently complicates distressed premature neonates in the first 10 days of life [1,2]. While it has been occasionally documented in older infants, it has rarely been reported as a postoperative sequela [2]. We here report a case of a 3-month-old infant who presented with a late onset NEC, possibly related to postoperative stress, that was complicated with colonic stricture. This is the first reported case of an infant experiencing a recurrence of NEC after herniotomy.

## Case presentation

A 3-month-old male infant was admitted to our pediatric department due to poor feeding and non-bilious vomiting for 3 days. He was born healthy at 36+2 weeks of gestation via C-section. The mother had COVID-19 infection twice during pregnancy and was successfully treated and recovered with no complications. His birth weight was 2.7 kg. The infant had been breastfed mainly with supplemental three times daily formula feeds. Family history was non-contributory.

At 10 days of age, he started to have loud bowel sounds, projectile vomiting, and irritability, and he rejected the formula feeds only. Cow's milk protein allergy (CMPA) and gastro-esophageal reflux (GERD) were suspected. He was started on CMP-free formula and esomeprazole elsewhere. No improvement was noticed.

At 20 days of age, he had an open bilateral inguinal herniotomy at another facility. After a few hours of the procedure, he developed rectal bleeding and abdominal distention. A gastrointestinal pathogens panel revealed norovirus and cryptosporidium. An abdominal ultrasound revealed portal vein gas but otherwise unremarkable findings. Bacterial translocation and NEC were suspected. Conservative treatment was initiated, and the infant relatively improved. Thus, he was discharged home. A few days later, the abdominal distention became worse, and the left inguinal hernia recurred. He had decreased feeding, recurrent episodes of whitish projectile vomit, infrequent loose stool, and poor weight gain. He was admitted six times to other facilities and then referred to our center.

At the time of admission, the infant was lethargic and dehydrated. Abdominal distention, increased abdominal sounds, mild generalized tenderness, and a partially reducible left inguinal hernia were noted on the physical examination. Blood tests showed raised platelets, monocytes, C-reactive protein, procalcitonin, and normocytic normochromic anemia as illustrated in Table 1.

**Table 1:** Blood test results; (H) = high, (L) = low.

Lab view	Result
White blood cells	15.9 *10^9/L
Red blood cells	3.64 cells/mcL
Hemoglobin	(L) 100 g/L
Hematocrit	0.302 L/L
Mean cell volume	83 Fl
Mean cell hemoglobin	28 picograms/cell
Mean cell hemoglobin concentration	331 g/L
Red cell distribution width	(H) 15.5%
Platelets	(H) 792 *10^9/L
Mean platelet volume	10.9 Fl
Neutrophil automated absolute	3.56 *10^3/UL
Lymphocyte automated absolute	9.88 *10^3/UL
Monocyte automated absolute	(H) 2.33 *10^3/UL
Eosinophils automated absolute	0.11 *10^3/UL
Basophils automated absolute	0.04 *10^3/UL
C-reactive protein	(H) 23 mg/L
Procalcitonin	(H) 0.11 ng/mL

**Figure 1:**
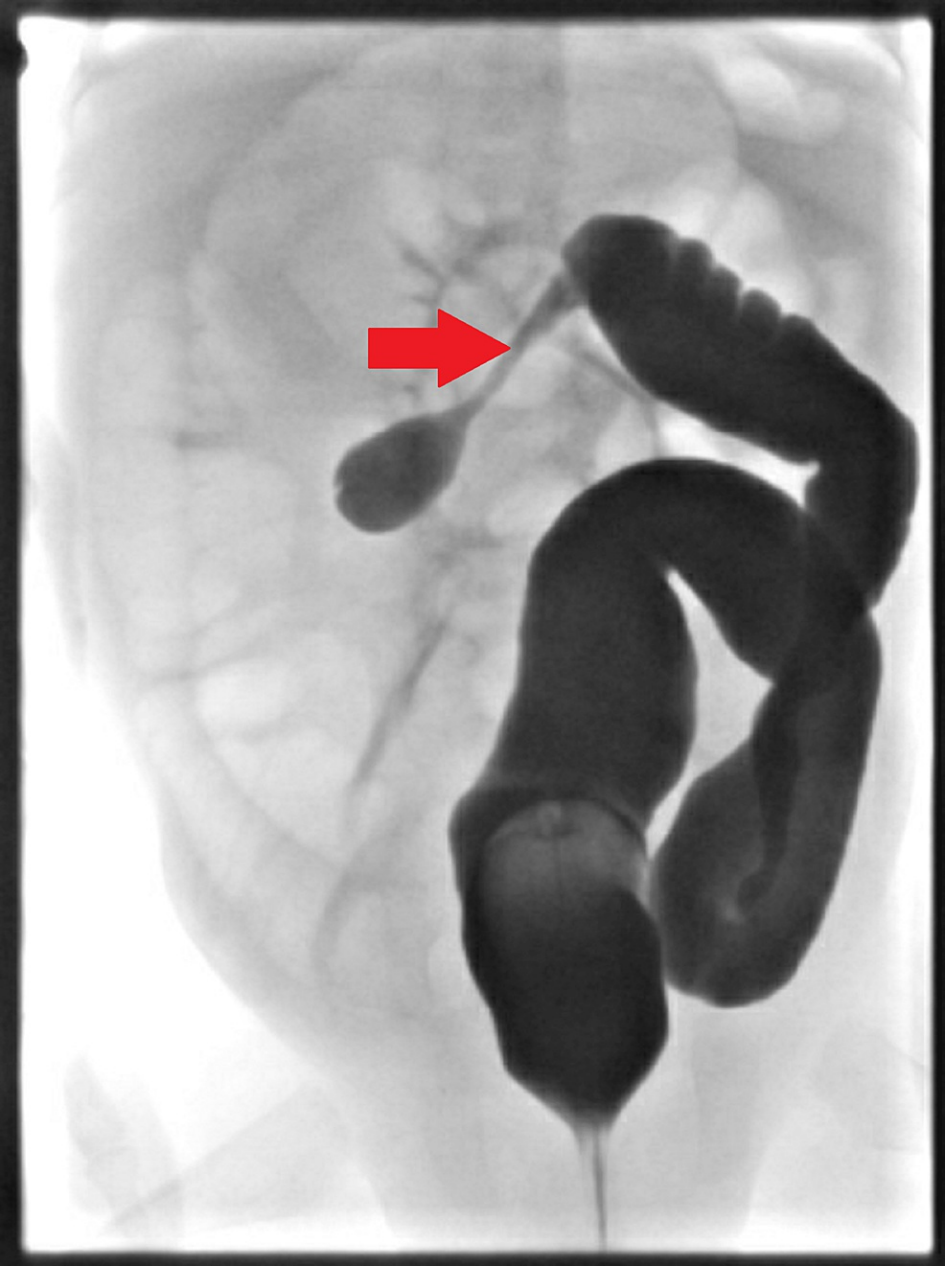
Barium enema showing a right-side transverse colon of a 3-month-old infant. The arrow indicates the stricture seen in the colon.

Contrast enema revealed significant narrowing of the right-middle transverse colon in keeping with stricture as illustrated in Figure 1. 

**Figure 2:**
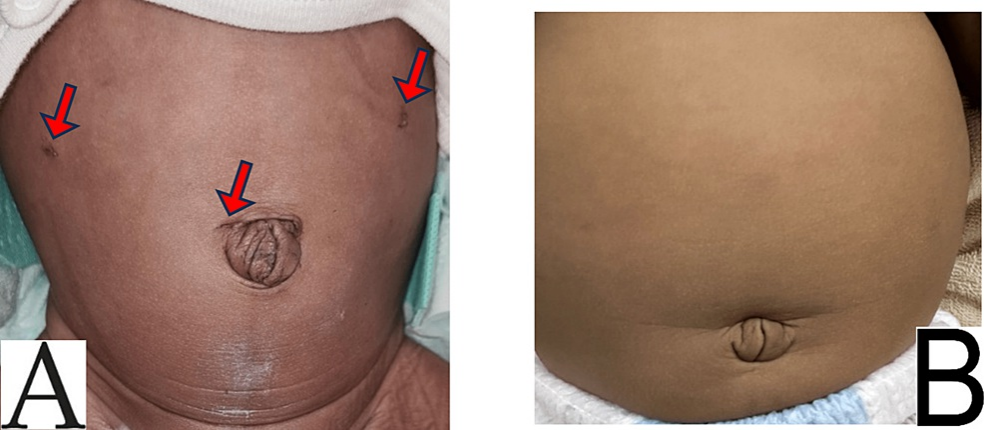
A: Laparoscopic entry incisions indicated by the arrows; B: Healed, scarless abdomen 7 months after the procedure.

The infant was taken to the operating room where rectal suction biopsies were performed and sent for a frozen section, followed by laparoscopic repair of the left recurrent inguinal hernia and a mini-laparotomy resection of the right-sided colonic stricture (6 cm length) with colo-colic anastomosis through a trans-umbilical incision. The frozen section showed a ganglionic bowel. The pathology of the resected right colonic stenosis showed segmental bowel wall fibrosis, loss of muscularis consistent with prior perforation, ulcerated mucosa with acute inflammation, serosal fibrovascular adhesions, and multiple benign reactive lymph nodes. The baby had an uneventful postoperative recovery and was discharged home after one week. He remains well 7 months after the procedure. Early (A), and late (B) postoperative appearance of the abdomen is shown in Figure 2.

## Discussion

Only a few cases of NEC due to postoperative noncardiac procedure-related stress have been reported in the literature. Comprehensive research was conducted in PubMed, Scopus, ScienceDirect, Embase, Web of Science, and Google Scholar from 1980 to date. Our review of the literature showed only 72 reported patients who had NEC after noncardiac surgeries, including myelomeningocele, intestinal atresia, gastroschisis, omphalocele, and congenital diaphragmatic hernia [2,4-10]. The details of our literature review are listed in Table 2. The development of postoperative NEC in inguinal hernia repair has been rarely mentioned. Of those 72 babies, only 5 had an inguinal herniotomy [5,6,8,10].

**Table 2:** Reported cases of NEC after noncardiac surgeries. NEC: Necrotizing enterocolitis.

Paper	Initial surgical intervention	Clinical manifestations	Imaging results specific to NEC	Comments
Amoury RA et al., 1980 [4]	Esophageal atresia (1), small intestinal atresia (3), anorectal atresia (1), gastroschisis (3)	Abdominal distension (in all), blood in the stool (in 3), loose stool (in 2), acidosis (in 1), jaundice (in 3)	Gas in the portal vein was found in 3 of the 9 babies, and pneumatosis intestinalis was found in all of them	Of a total of 9 patients, 6 died mainly of sepsis
Daniel L et al., 1982 [2]	Malrotation (1), aganglionic megacolon (1), gastroschisis (7), omphalocele (1), jejunal atresia (1), Ileal atresia (1)	Abdominal distension (in 7), lethargy (in 2), vomiting (in 4), blood in the stool (in 5), abdominal cellulitis (in 2), acidosis (in 1), jaundice (in 1)	Seven infants underwent radiographic examination, and all demonstrated pneumatosis intestinalis and/or portal vein air	Of a total of 11 patients, 5 died mainly of sepsis, and 3 had recurrent NEC
Oldham KT et al., 1988 [9]	Gastroschisis (10)	All the patients had: abdominal distension, vomiting, blood in the stool	Pneumatosis intestinalis	Of a total of 10 patients, 2 died, and all had recurrent NEC
Shanbhogue LKR et al., 1991 [5]	Spina bifida (12), hydrocephalus (5), diaphragmatic hernia (5), intestinal atresia (2), anorectal anomaly (2), gastroschisis (2), esophageal atresia (2), inguinal hernia (2)	Each of the two recurrent episodes of NEC presented with rectal bleeding without systemic signs of infection	Pneumatosis of the bowel existed in the two recurrent episodes of NEC	Of a total of 33 patients, 10 died of sepsis or pneumonia, and 2 had recurrent NEC
Jayanthi S et al., 1998 [7]	Gastroschisis (8)	All the patients had: abdominal distension, vomiting, blood in the stool	Pneumatosis intestinalis was found in 7 patients, and gas in the portal; vein was found in 1	A total of 8 patients survived
Türkyilmaz Z et al., 2001 [6]	Inguinal hernia (1)	Abdominal distension, vomiting, acidosis, loose stool	Pneumatosis intestinalis was found	Died of sepsis
Kumar C et al., 2022 [10]	Inguinal hernia (1)	Abdominal distension, vomiting, blood in the stool, acidosis	Pneumatosis intestinalis	Died due to unknown cause
Prasad G et al., 2023 [8]	Inguinal hernia (1)	Abdominal distension, vomiting, blood in the stool, acidosis	N/A	Survived
Our case, 2023	Inguinal hernia (1)	Abdominal distension, vomiting, blood in the stool	Gas in the portal vein	Survived

Our patient's clinical and radiological features were suggestive of NEC both before and after the first surgery. However, the infant improved with supportive therapy. We propose that the bowel was prone to inflammation, necrosis, and stricture formation due to formula feeding, previous NEC, and surgical stress. Amoury et al. related NEC to surgical stress caused by hypoperfusion resulting from increments in intraluminal pressure that lead to mucosal injury [4]. In our case, the repair of bilateral hernias and reperfusion of the colonic segments might have triggered the inflammatory cascade and changed the intraluminal pressure, which facilitated the recurrence of NEC. Moreover, Pelizzo et al. suggested that norovirus infection increases the mucosal affinity for severe, distinctive colonic ischemic lesions in infants [11], which might have contributed to our case presentation.

All the previous papers highlighted abdominal distension, vomiting, and bleeding per rectum as demonstrations of NEC in babies at a median of 3 days postoperatively, all of which were encountered in our setting. The distinctive radiologic signs of NEC are pneumatosis intestinalis (air within the wall of the intestine), gas in the portal vein, pneumoperitoneum, and dilated loops of the bowel [1,2,4,9]. Nevertheless, portal venous gas is not commonly present and is a poor prognostic sign if detected [1], which was the case here.

Overall mortality might reach 50% [1]. For infants who have full-thickness necrosis of the intestinal wall leading to perforation and peritonitis, the mortality rate could approach 100% [1]. A significant number of reported postoperative NEC cases ended up with sepsis and death [2,4-6,9].

Food protein-induced enterocolitis syndrome (FPIES) is a non-IgE-mediated allergic reaction typically triggered by cow's milk and soy protein. In neonates, it manifests as persistent emesis within 1-3 hours after feeding, accompanied by other symptoms such as lethargy, diarrhea, bloody stool, abdominal distension, anemia, eosinophilia, metabolic acidosis, and failure to thrive [12,13]. Notably, these symptoms mirror those of NEC, leading to diagnostic and therapeutic pitfalls. However, FPIES rarely exhibit abdominal tenderness or sepsis, and neonatal fatalities are uncommon [13]. Furthermore, our case notably lacks the characteristic eosinophilia typically associated with FPIES, and he did not show improvement on the CMP-free formula.

The combined laparoscopic and mini-laparotomy approach in our case led to ease in repairing the recurrent hernia by accessing it from a virgin area, and to optimal cosmetic results given the complex, multiple procedures he had concurrently. The resulting scars are almost invisible. We recommend that recurrent hernias are approached laparoscopically whenever possible even in the context of the need for simultaneous open surgery. The trans-umbilical incision is quite versatile in infants and can be used even for extensive bowel resection with excellent results [14].

Though recurrence of postoperative NEC has been reported [2,5,9], this case represents the first documented instance of an infant experiencing a recurrence of NEC with stricture possibly resulting from herniotomy. Our case signifies the challenges in diagnosing and managing postoperative NEC after herniotomy. It is particularly noteworthy that NEC can occur in full-term, healthy, breastfed infants of a normal weight.

## Conclusions

This case highlights the importance of considering postoperative stress as a causative factor for complicated NEC, even when infants presents with the clinical and radiological manifestations of NEC after simple procedures such as inguinal hernia repair. Healthcare providers should have high index of suspicion to avoid devastating consequences.
